# Impact of Diverse Nanostructure Forms of NiCo_2_O_4_ Bulk Ceramics on Electrical Properties

**DOI:** 10.1021/acsomega.5c00708

**Published:** 2025-04-22

**Authors:** Orhun Dos, Sukru Cavdar

**Affiliations:** † Department of Advanced Technologies, Graduate School of Natural and Applied Sciences, Gazi University, Ankara 06530, Turkey; ‡ Alparslan Defence Sciences and National Security Institute, National Defence University, Ankara 06530, Turkey; § Department of Physics, Faculty of Science, Gazi University, Ankara 06530, Turkey

## Abstract

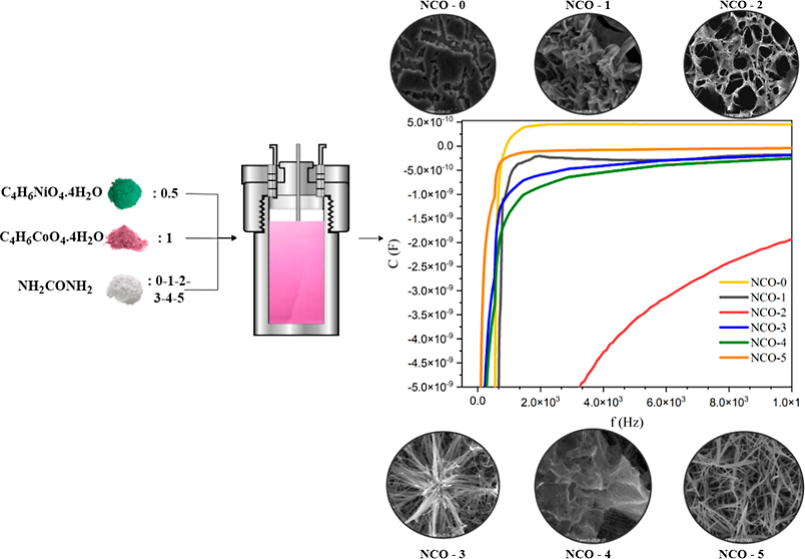

Spinel NiCo_2_O_4_ is a promising material for electronic applications due to its tunable nanostructures and electrical properties. This study systematically investigates the effect of varying urea concentrations in the hydrothermal solution on the nanostructural evolution of NiCo_2_O_4_ (NCO) and its impact on electrical and dielectric properties. By varying urea content, distinct morphologies—including nanosheets, nanoleaves, and nanofibers—were obtained and characterized via XRD, XPS, and FE-SEM analysis. To evaluate the electrical characteristics, capacitance and conductance measurements were performed using an LCR Meter at room temperature over a frequency range of 100 Hz to 1 MHz. The results revealed negative capacitance at low frequencies, linked to polarization changes and minority charge carriers. Also, dielectric parameters were calculated from measured values, showing that nanoform variations influence conductivity trends. This study provides new insights into how nanoform variations in NiCo_2_O_4_, controlled by synthesis parameters, influence its dielectric behavior. The findings significantly contribute to the understanding of negative capacitance and the tunability of electrical properties in NCO, highlighting their potential for future electronic and dielectric applications.

## 1 Introduction

Nanomaterials exhibit exceptional physical, chemical, and electrical properties that depend on their structure, form, and size. Consequently, recent studies have focused on developing new nanostructures with controllable features to optimize these properties.^1,2^ Spinel oxides (AB_2_O_4_), a unique class of materials, are widely used in next-generation applications due to their diverse structural, chemical, and electrical properties.^3,4^ Transition metal oxides are synthesized using various chemical and physical methods. They are valued for their high conductivity, specific capacitance, surface reactivity, environmental friendliness, affordability, and excellent capacitive properties.^5,6^ NiCo_2_O_4_ (NCO), a transition metal oxide of the AB_2_O_4_ spinel type, is distinguished by its favorable properties in related research.^7−9^ Superior electrochemical activity and electronic conductivity compared to NiO and Co_3_O_4_ are exhibited by spinel NCO, offering significant advantages for practical applications.^10,11^

To date, extensive research has been conducted to synthesize various NCO nanostructures, including nanosheets,^12^ nanoflakes,^13^ nanorods,^14^ nanowires,^15^ nanoneedle,^16^ nanospheres,^17^ and nanoflowers.^18^ Several processing techniques, such as hydrothermal,^19^ coprecipitation,^20^ sol–gel,^21^ and electrodeposition^22^ have been employed to produce NCO with diverse morphologies.

Literature studies have highlighted the role of urea in promoting the homogeneous precipitation of chemical precursors in hydrothermal solutions.^23,24^ Urea also serves as a critical control parameter in determining the morphology of NCO nanoparticles during hydrothermal synthesis.^25,26^ Research has shown that increasing the amount of urea leads to the formation of finer nanoparticles with gradually increasing surface area in the final product.^27^ Additionally, NCO can be synthesized in a porous nanostructured form by optimizing hydrothermal parameters, making it suitable for electrical applications.^28^ Given the growing demand for materials with high surface area in small volumes, NCO stands out as a promising transition metal oxide in this domain.

This study stands out by providing a detailed investigation of how different nanoforms of NCO, synthesized using varying molar ratios of urea in the hydrothermal process, affect the material’s electrical properties. Based on the analysis of previous studies, the hydrothermal method was selected for the synthesis of NiCo_2_O_4_ nanostructures due to its reliability and the extensive range of controllable parameters it offers. As one of the key parameters, NCO was synthesized in different nanostructure forms by varying the amount of urea added to the hydrothermal solution. The resulting samples were then subjected to a series of tests and analyses to assess their structural, chemical composition, morphology, and electrical properties. The main aim of this study is to examine the negative capacitance and dielectric behavior of NCO, with a particular focus on their dependence on the various nanostructure forms of NCO. Field emission scanning electron microscopy (FE-SEM) imaging was used to characterize the nanostructure form of the final sample, which was influenced by variations in urea concentration, while its structural properties were determined using Debye–Scherrer analysis of X-ray diffraction (XRD) data, and its surface chemical composition was analyzed through X-ray Photoelectron Spectroscopy (XPS). The capacitance and conductance of the NCO samples were experimentally measured by LCR Meter at room temperature across a frequency range of 100 Hz to 1 MHz. The impact of morphological variations in the NCO samples on their dielectric behavior was thoroughly analyzed and discussed.

## 2 Experimental Section

### 2.1 Materials Required

The chemical precursors used in the experiment were Cobalt acetate tetrahydrate (C_4_H_6_CoO_4_·4H_2_O—Alfa Aesar, Massachusetts, United States of America, %98), nickel acetate tetrahydrate (C_4_H_6_NiO_4_·4H_2_O—Central Drug House, New Delhi, India, %98), Urea (CH_4_N_2_O—Isolab Chemicals, Wertheim, Germany, 99.0%), Ultra Pure Distilled Water (UPDW—Elga Purelab Classic UVF, Celle, Germany).

### 2.2 Synthesis of NiCo_2_O_4_ Nanoparticles by Hydrothermal Method

**Figure 1:**
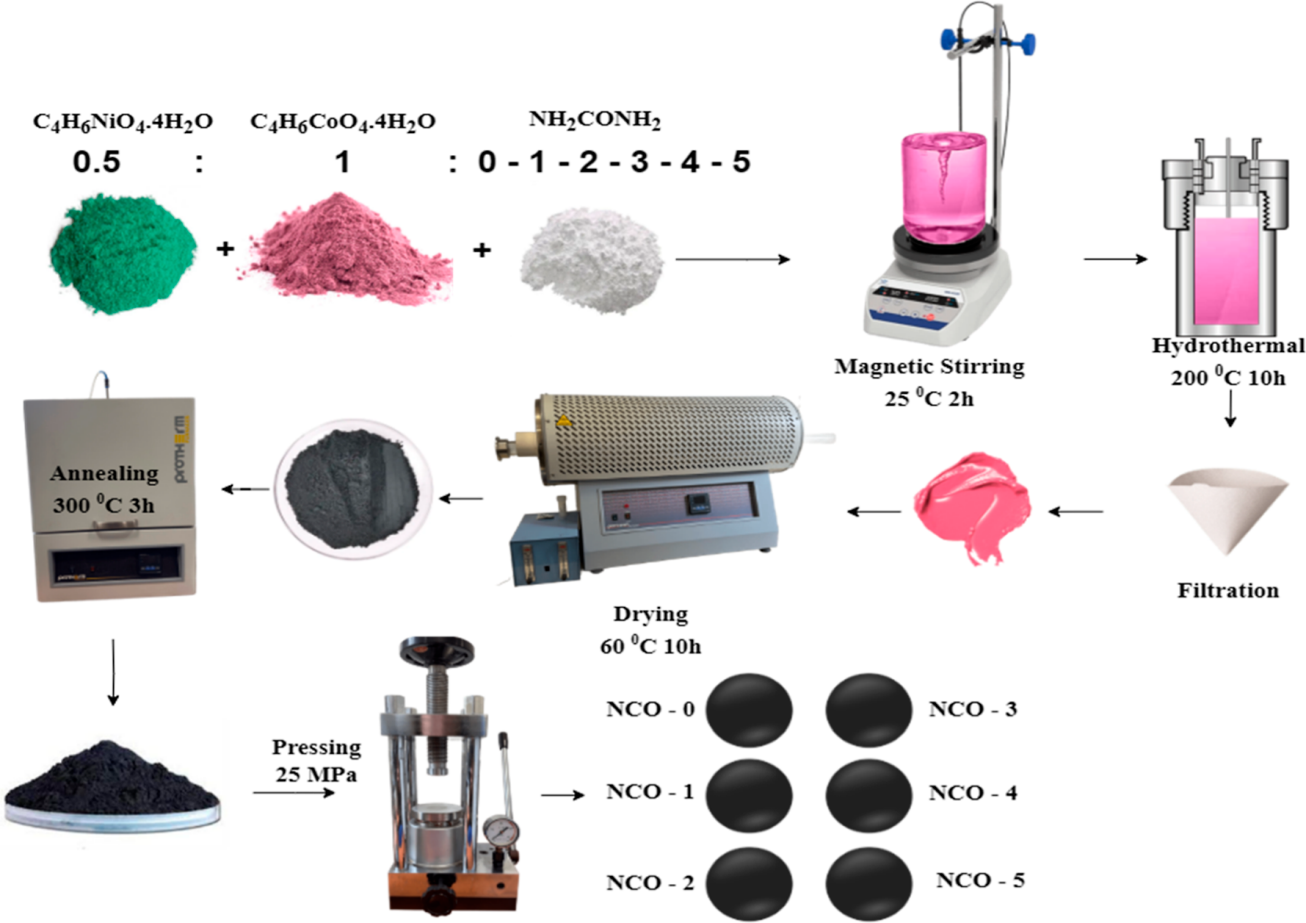
Experimental scheme of NCO production in hydrothermal method.

The hydrothermal method was selected for the synthesis of NiCo_2_O_4_ nanostructures due to its efficiency in controlling morphology. Ni^+^ and Co^+^ ions required for the synthesis were chemically dissociated from their acetate precursors, nickel acetate tetrahydrate (C_4_H_6_NiO_4_4H_2_O) and cobalt acetate tetrahydrate (C_4_H_6_CoO_4_4H_2_O), respectively. Urea (CH_4_N_2_O) was introduced into the solution as a morphological control agent, influencing the pH of the solution in molar ratios specified in Table 1. The synthesized samples were designated as NCO-0, NCO-1, NCO-2, NCO-3, NCO-4, and NCO-5, corresponding to the urea molar ratios. The precursors were combined in the molar ratios outlined in Table 1 and dissolved in 50 mL of ultrapure distilled water. The solution was stirred at room temperature (25 °C) for 2 h using a magnetic stirrer (WN-H320, Weightlab, Istanbul, Türkiye), resulting in a homogeneous light pink solution. The prepared solution was transferred to a Teflon-lined container and subjected to a hydrothermal reaction in a stainless-steel autoclave at 200 °C for 10 h with the temperature incrementally increased by 5 °C per minute. Upon completion of the reaction, the autoclave was allowed to cool naturally to room temperature. The NCO particles, which had precipitated to the bottom of the solution, were separated from the water solvent using filter paper. The resulting precipitate, which exhibited a dark pink sludge-like consistency and retained water molecules, was dried in a dry N_2_ atmosphere within a tube furnace (PZF 12/105/900, Protherm, Ankara, Türkiye) at 60 °C for 10 h, producing a black powder. Following the drying process, the sample was annealed in a furnace (PLF 160/5, Protherm, Ankara, Türkiye) at 300 °C for 3 h, with the temperature incrementally increased by 1 °C per minute. This annealing step enhanced the crystallographic structure of the NCO and removed residual organic components through combustion. The annealed sample was then compacted into bulk form using a hydraulic pellet-pressing machine under a pressure of 25 MPa at room temperature, rendering it suitable for electrical measurements. The entire experimental procedure, as illustrated in Figure 1 was repeated six times with varying urea concentrations to investigate the effect of urea content on the properties of the synthesized material.

**Table 1:** Design of Experimental Parameters

sample name	molar ratio Ni/Co/urea	hydrothermal temperature (°C), time (h) and ramp rate (°C/m)	annealing temperature (°C), time (h) and ramp rate (°C/m)
NCO-0	0.5:1:0	200 °C, 10 h, 5 °C/m	300 °C, 3 h, 1 °C/m
NCO-1	0.5:1:1	200 °C, 10 h, 5 °C/m	300 °C, 3 h, 1 °C/m
NCO-2	0.5:1:2	200 °C, 10 h, 5 °C/m	300 °C, 3 h, 1 °C/m
NCO-3	0.5:1:3	200 °C, 10 h, 5 °C/m	300 °C, 3 h, 1 °C/m
NCO-4	0.5:1:4	200 °C, 10 h, 5 °C/m	300 °C, 3 h, 1 °C/m
NCO-5	0.5:1:5	200 °C, 10 h, 5 °C/m	300 °C, 3 h, 1 °C/m

In this study, the transition between the nanoforms of the NCO products was regulated by varying the amount of urea in the hydrothermal solution, which served as the sole adjustable parameter throughout the experimental process. The synthesis procedure, schematized in detail in Figure 1, is described by chemical reaction equations in eqs 1–4. Equations 1–3 depict the decomposition processes of nickel acetate, cobalt acetate, and urea, respectively. Equation 4 outlines the synthesis of NiCo_2_O_4_, where Ni^2+^ and Co^2+^, Co^3+^ ions react with urea-derived intermediates, accompanied by the release of gaseous byproducts. During the drying stage, water molecules were eliminated, and in the subsequent calcination stage, organic residues originating from urea were removed, resulting in the crystallized final product.
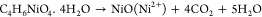
1
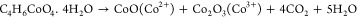
2
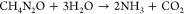
3
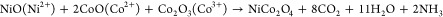
4

### 2.3 Materials Characterization

As outlined in Section 2.2, multiple analyses were conducted to evaluate the structural, chemical, morphological, and electrical properties of the synthesized NCO samples. To determine the crystallographic structure, XRD analysis (Bruker D8 Advance, Massachusetts, USA) was performed using the powder technique with a Cu–Kα source (wavelength: 1.54056 Å). Measurements were conducted over a 10° to 80° range at a scanning speed of 2° per minute. XPS analysis was performed to determine the binding energies of elements and reveal the surface chemistry of the synthesized NCO samples. The measurements were conducted using the PHI 5000 VersaProbe device with an Al monochromatic X-ray anode and an argon ion gun, operating at an etching rate of 2 keV. FE-SEM analysis was conducted to examine the effect of varying urea ratios on NCO nanoforms synthesized via the hydrothermal method. Images of NCO samples were captured at a 2 μm scale with a resolution of 9.9 mm × 25k at 5 kV in a 200 Pa vacuum using the HITACHI SU8700 device. EDS-MAP analysis was performed to determine the elemental distribution of Ni, Co, and O on the sample surfaces. The analysis was conducted using the Oxford Ultim-Max 100 EDS detector (100 mm^2^ detector area) integrated with the HITACHI SU8700 device. Electrical characterization involved capacitance and conductance measurements, performed at room temperature across a frequency range of 100 Hz to 1 MHz using a Sourcetronic ST2826 LCR Meter.

## 3 Result and Discussion

### 3.1 Structural Analysis

**Figure 2:**
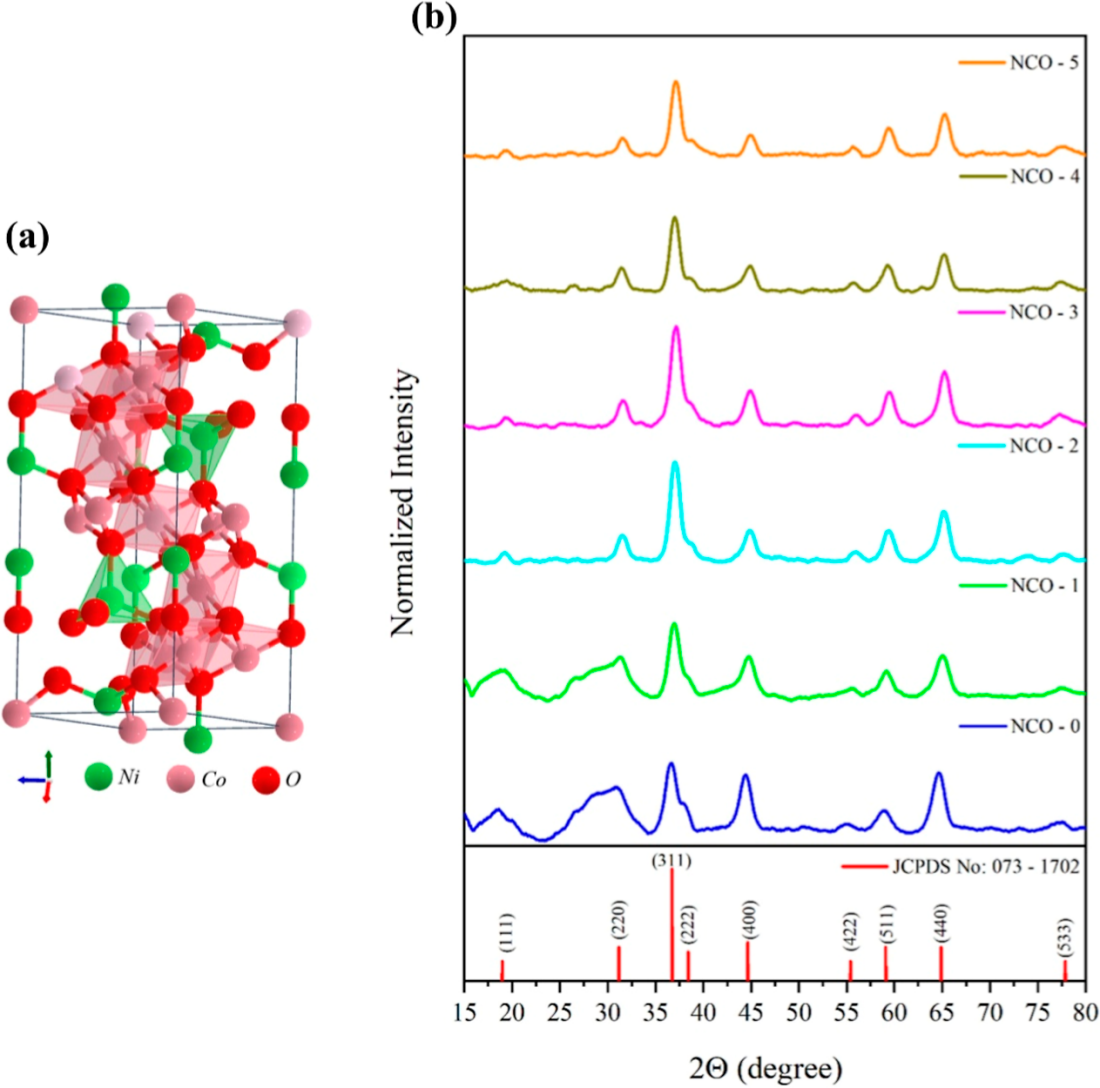
(a) Crystal structure of NCO,^33^ (b) XRD pattern of NCO nanostructures.

Figure 2 illustrates the XRD analysis results, demonstrating that all NCO samples synthesized in this study align with the crystallographic pattern of NiCo_2_O_4_, as specified in JCPDS no: 073-1702, characterized by nine distinct diffraction peaks.^29^ The detected peaks were located at 2θ values of 18.9°, 31.2°, 36.7°, 38.4°, 44.8°, 55.4°, 59.1°, 64.9°, and 76.1°, corresponding to the (hkl) planes of (111), (220), (311), (222), (400), (422), (511), (440), and (533), respectively. These peaks confirm that the spinel NiCo2O4 structure exhibits a cubic phase. Also, Figure 2 illustrates the final lattice structure of NCO crystals following thermal annealing (calcination), a process that enhances the crystal architecture of NiCo_2_O_4_. The detailed analysis and insights gained from the previous study^30^ on the NCO-0 sample that synthesized without urea. Based on this, the NCO-0 sample was chosen as a reference in the current study to evaluate the effect of urea. When the XRD patterns of the NCO samples are normalized and presented in Figure 2, it is evident that the peaks below 35° in 2θ for NCO-0 (synthesized without urea) and NCO-1 (synthesized with the lowest urea content) exhibit significantly broad diffraction patterns, indicative of crystal defects.^31,32^ However, with all experimental parameters held constant, an improvement in peak intensities is observed in the crystal structures of the samples from NCO-2 to NCO-5, correlating directly with the increasing urea content in the synthesis process. Additionally, the absence of impurity peaks in the XRD patterns confirms the high purity of the acetate precursors used in the synthesis.

The crystal size (*D*), microstrain (ε), interplanar distance (*d*_hkl_), and lattice constant (*a*) values calculated from the XRD results provided detailed insights into the crystallographic properties of the NCO samples. The XRD parameters presented in Table 2 were calculated using equations derived from Scherrer’s formula^34^ and Bragg’s law.^35^ The symbols *D*, *K*, λ, β, θ, *d*, and *n* represent the following: crystallite size (*D*), shape factor (*K*, typically assumed to be 0.9 for a cubic lattice), wavelength (λ, specifically 1.54056 Å), full width at half-maximum (fwhm, β), peak position (θ), interplanar distance (*d*), and diffraction order (*n*), respectively. An increase in crystal size was observed with higher urea content, suggesting that the amount of urea in the synthesis solution plays a critical role in the nucleation process and influences the growth kinetics of the crystals.^36^ Microstrain values, derived from the broadening of the XRD diffraction peaks, reflect the deformations within the NCO crystal structure. The results indicate that the NCO-4 and NCO-5 samples, synthesized with the highest urea content and exhibiting the lowest microstrain values, possess a more uniform crystalline order. This can be attributed to reduced internal stresses in these samples.^37^

**Table 2:** XRD Parameters of NCOs with Different Nanoforms

	crystal size *D* (nm)	microstrain ε × 10^–3^	interplanar distance (*d*_hkl_)	lattice constant *a* (Å)
formula	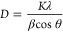	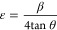	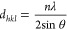	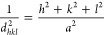
sample name				
NCO-0	5.82	2.18	2.44	8.12
NCO-1	6.73	1.90	2.42	8.05
NCO-2	6.74	1.90	2.42	8.04
NCO-3	6.23	2.06	2.41	8.01
NCO-4	7.35	1.74	2.42	8.04
NCO-5	6.90	1.86	2.41	8.01

The interplanar distances reveal the effect of urea on the unit cell dimensions of the crystal NCO structure. These distances exhibit very similar values across all samples, indicating a consistent crystallographic order and a reliable experimental systematic in the hydrothermal synthesis method.^38^ The lattice constant, representing the unit cell edge length in the cubic crystal structure of spinel NCO, was found to be nearly identical to the literature-reported value of 8.11 Å.^39^ This suggests that urea, despite being the only variable in the synthesis process, does not significantly influence the cubic structure of the material.

Overall, the XRD results suggest that the hydrothermal synthesis method allows for the facile synthesis of diverse nanomaterials for multiple transition metal oxides. This can be achieved by simply adjusting the amount of urea in the molar ratios of the transition metal precursors.^25,40−43^

### 3.2 Chemical Composition Analysis

**Figure 3:**
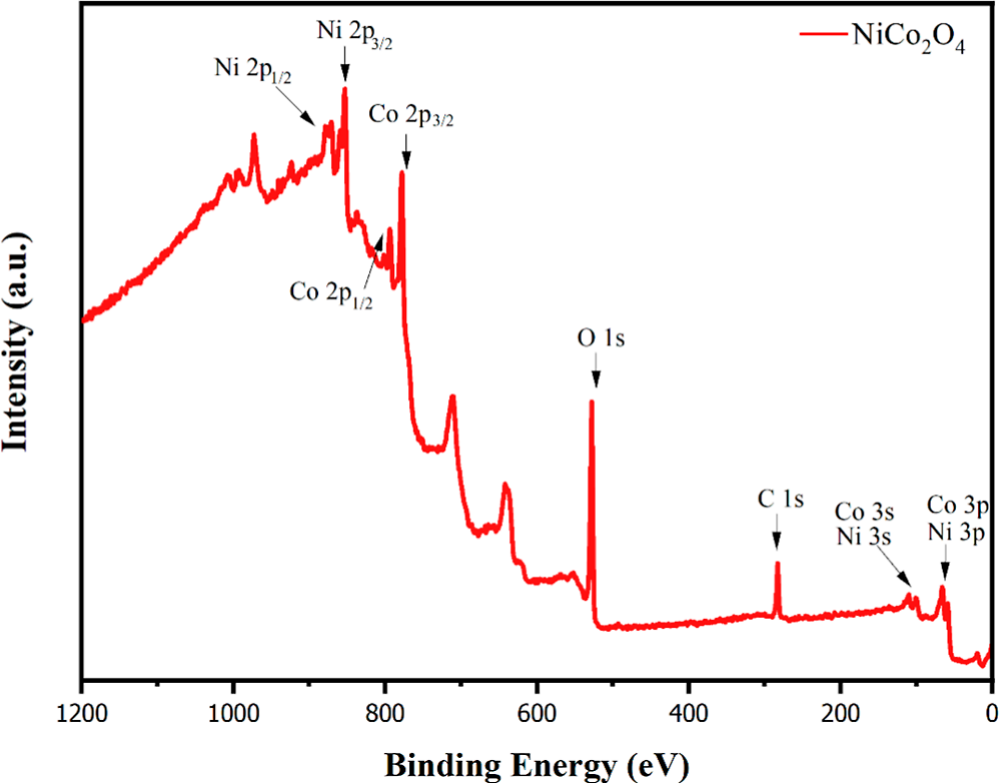
XPS survey spectrum of NCO.

To determine the elemental composition and surface chemistry of the synthesized NCO samples, XPS analysis was conducted to provide detailed insights into the chemical binding energies of the surface elements. The binding energy intensities, as presented in Figure 3, were analyzed. The observed elements and their binding energy intensities were consistent with the XRD results and corroborated by findings reported by other research groups working in this domain.^44,45^

**Figure 4:**
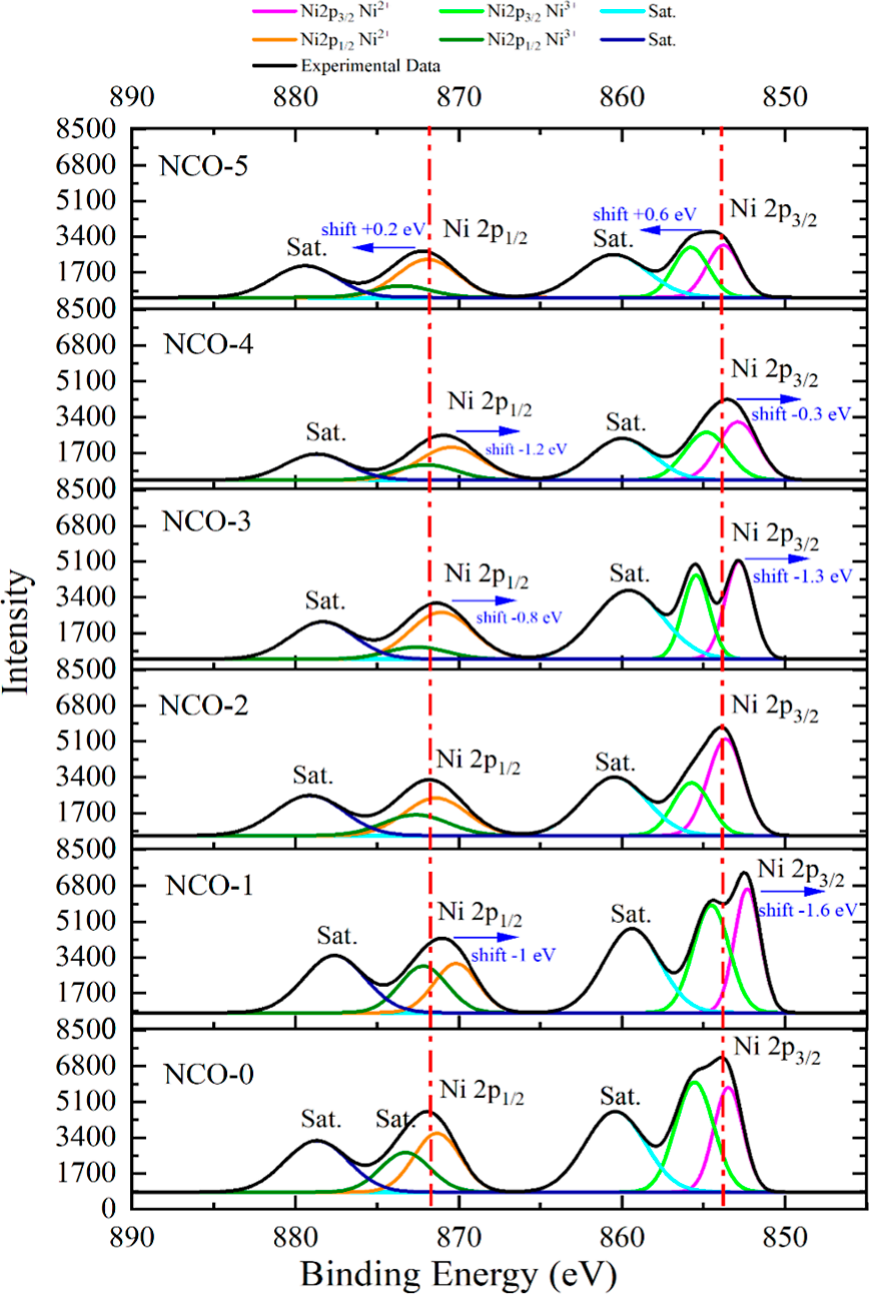
XPS core level spectra of Ni 2p.

In the high-resolution spectra, with NCO-0 serving as the reference for comparison with other NCO samples, Ni 2p peaks are observed within the binding energy range of 890 to 850 eV, with intensities reaching up to 8500, as shown in Figure 4. Ni 2p peaks correspond to the binding energies of electrons in the 2p orbitals of Ni^+^ ions. The Ni 2p, Ni 2p_3/2_ and satellite peaks detected in NCO-0 are positioned at binding energies consistent with values reported in the literature.^46^ This study examines the influence of urea on the oxidation state of Ni ions by analyzing the shifts in the positions of these characteristic peaks. Accordingly, an increase in urea content resulted in a reduction in the density corresponding to the binding energy of Ni 2p, attributed to the diminished surface concentration of Ni in the final sample. It is hypothesized that atoms such as C and N, originating from the decomposition of urea in the solution, obstruct the XPS signal of Ni. Moreover, the negative shifts in the binding energy peak values observed up to NCO-4 suggest a reduction in the oxidation state, likely due to an increase in electron density around the atoms.^47^ Conversely, the behavior observed in the NCO-5 sample indicates an increase in the oxidation state, as the atomic nucleus exerts a stronger attraction on electrons, leading to a tendency for the atom to become positively charged.^48^

**Figure 5:**
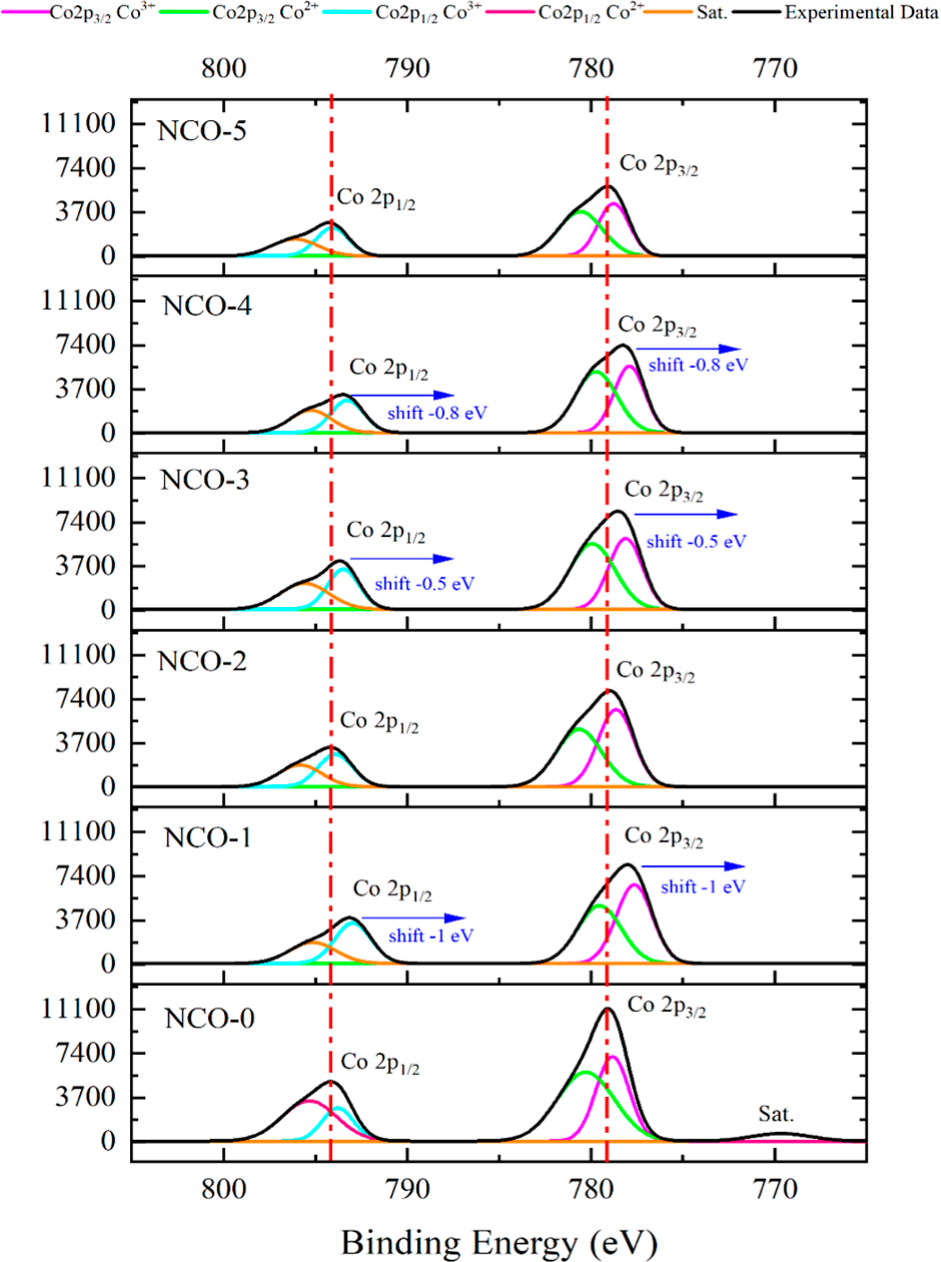
XPS core level spectra of Co 2p.

The Co 2p peaks, distributed at binding energies between 810 and 775 eV in the high-resolution XPS analysis, are shown in Figure 5, with NCO-0 referenced at an intensity of 11,100. The Co 2p_1/2_, Co 2p_3/2_ and satellite peaks in NCO-0 were observed at binding energy positions consistent with those reported in the literature.^49^ Similar to the Ni 2p XPS graph, a reduction in the XPS signal intensity of Co 2p and shifts in the binding energy positions of Co 2p peaks were observed as the urea content increased, particularly toward NCO-5. These shifts, occurring in the negative eV direction, are attributed to a decrease in the oxidation state.^50^ Furthermore, the satellite peaks appeared dampened relative to the *x*-axis due to the normalization of the intensity axis.

**Figure 6:**
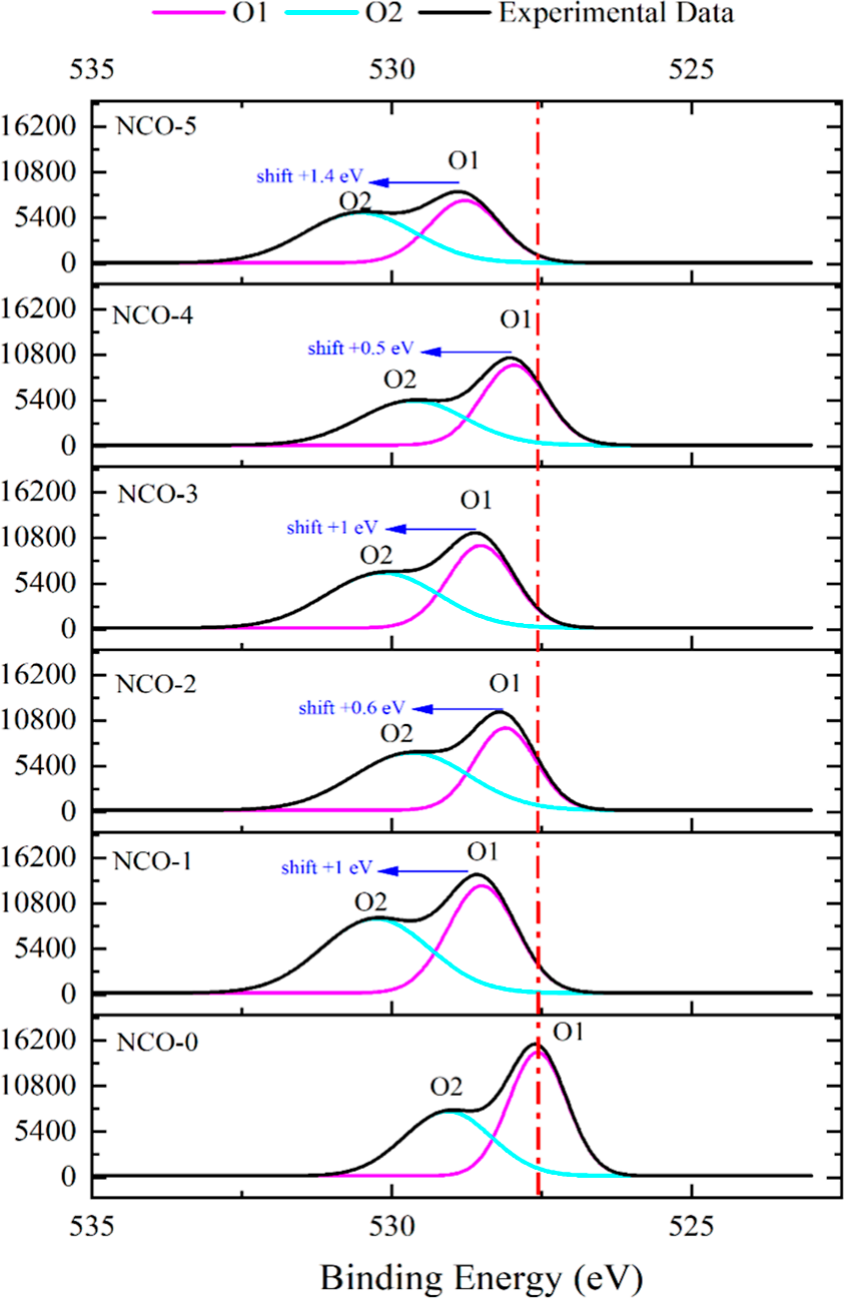
XPS core level spectra of O 1s.

The XPS analysis of O 1s, which displays two distinct peaks at binding energy positions between 535 and 525 eV, normalized to an intensity of 16,200, is presented in Figure 6. The O1 and O2 peak positions correspond to metal–oxygen bonding on the surface of NCO samples and the presence of oxygen vacancies and surface hydroxyl groups.^51^ The O 1s peak at approximately 531.5 eV suggests the presence of carbonate and oxycarbonate species, commonly observed on metal oxide surfaces.^52,53^ With increasing urea concentration, shifts in the positive eV direction were observed in all samples up to NCO-5. These shifts may suggest that the bonding between oxygen ions and metal ions is strengthened or that oxygen is present in a higher oxidation state.^54^

### 3.3 Morphological Analysis

**Figure 7:**
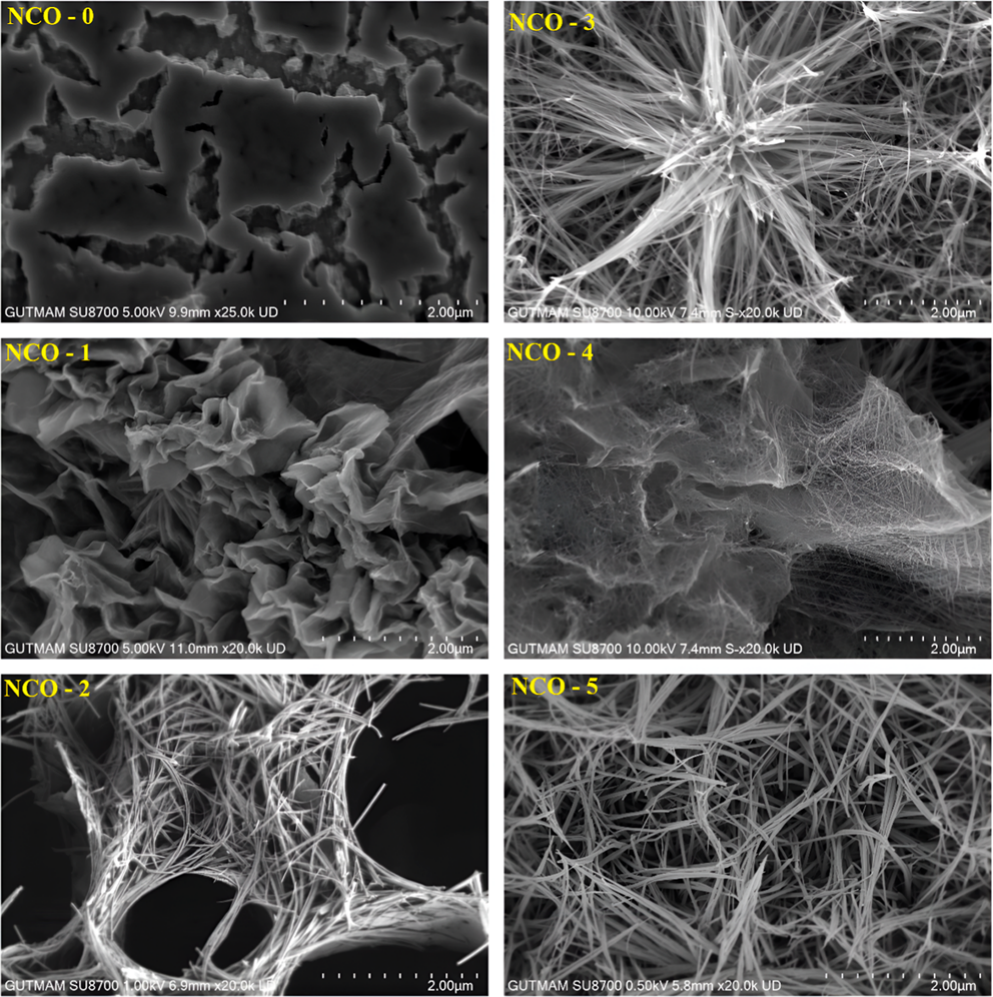
FE-SEM images of NCO nanostructures.

To analyze the hypothesis underlying this study—the effect of NCOs with different nanoforms on electrical properties—the influence of urea, selected as the variable parameter in the hydrothermal reaction, on the nanoforms of the final samples was first investigated. The morphological properties of the synthesized NCO nanomaterials, depending on their forms, were evaluated using FE-SEM images presented in Figure 7 at a 2 μm scale.

Accordingly, the effect of urea content on the nanoform of NCO was demonstrated, aligning with findings from independent studies in literature.^55,56^ It was observed that the NCO-0 sample exhibited nanosheet-like structures, NCO-1 had nanoleaf-like structures, NCO-2 displayed fiber mesh-like structures with spider nest-like hollows, NCO-3 featured chestnut-like structures with fibers, NCO-4 showed tulle-like structures with fibers, and NCO-5 presented a dense fiber mesh-like morphology. Additionally, all NCO samples were found to be randomly oriented in various regions. In general, given the critical role of effective surface areas in nanotechnology, this study aims to comparatively investigate the impact of different nanoforms on the electrical parameters of the samples.

**Figure 8:**
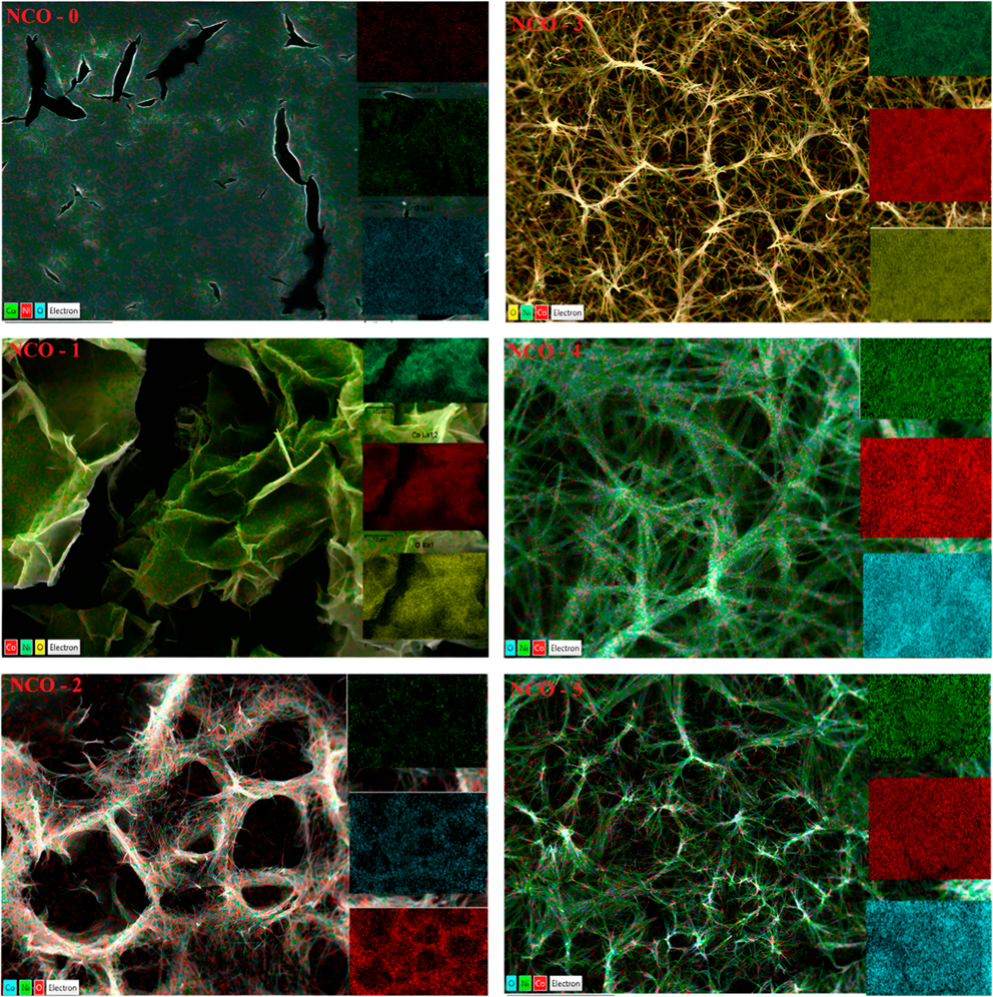
Elemental mapping images of NCO nanostructures.

The elemental mapping of NCO samples synthesized in various nanoforms, obtained through EDX analysis, is presented in Figure 8. Examination of the images captured at a 2.5 μm scale reveals that only Ni, Co, and O elements are present in the EDX spectrum, and these elements are distributed homogeneously. This indicates the absence of impurity elements in the NiCo_2_O_4_ structure. The findings confirm that NiCo_2_O_4_ can be synthesized in distinct nanoforms by regulating the amount of urea in the hydrothermal synthesis process, aligning well with the XRD data. Furthermore, it is assessed that the distribution of Ni, Co, and O in the samples may influence their electrical properties.^57,58^

An increase in the urea concentration within the solution during the hydrothermal synthesis process is hypothesized to influence the structure of NCO by primarily affecting the grain boundaries. This, in turn, may alter the growth dynamics of nucleation centers, leading to variations in cluster size and indirectly contributing to the differentiation of the nanoform.^55,59^

### 3.4 Electrical Analysis

**Figure 9:**
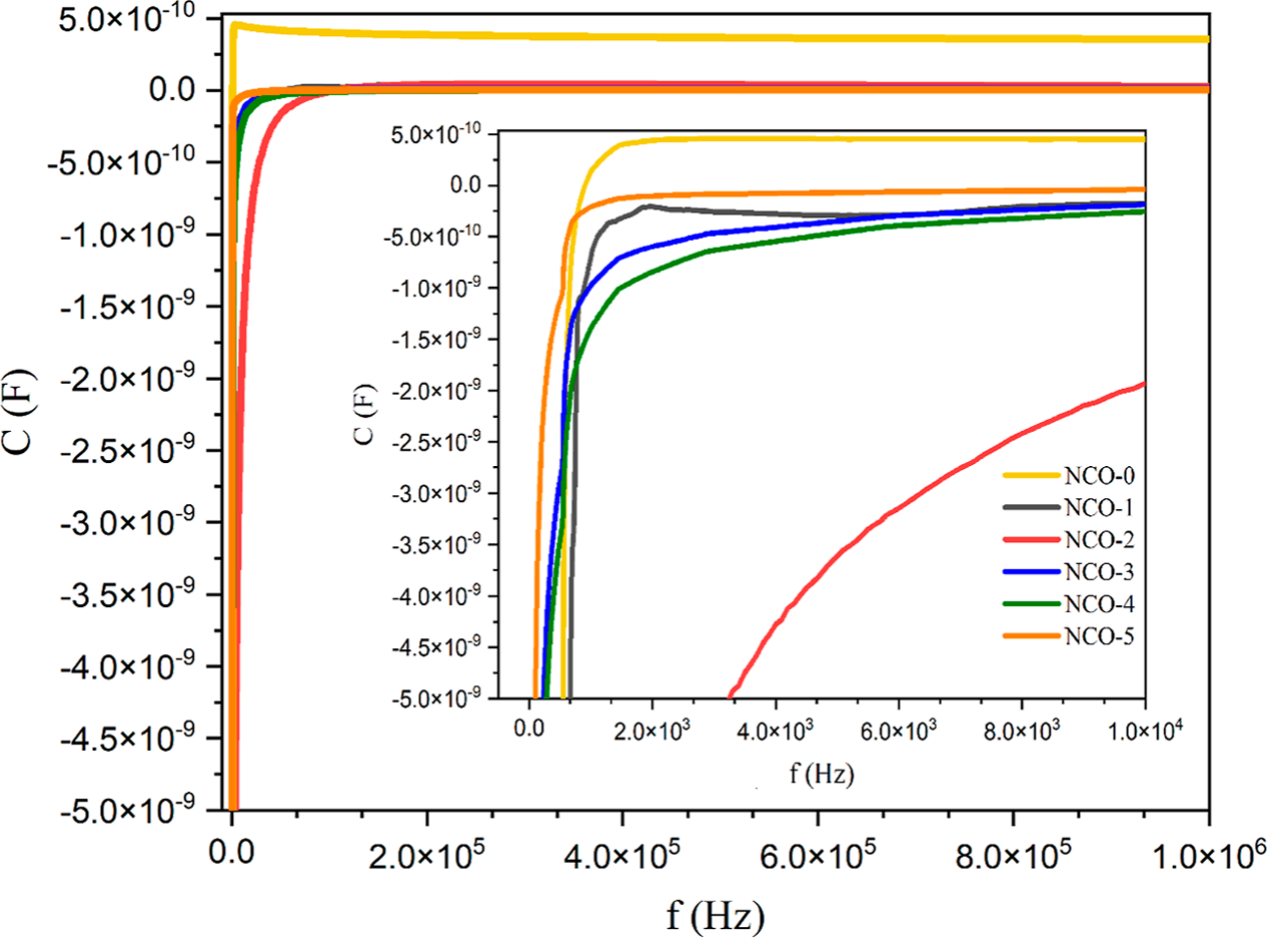
Capacitance versus frequency plot of NCO nanostructures.

**Figure 10:**
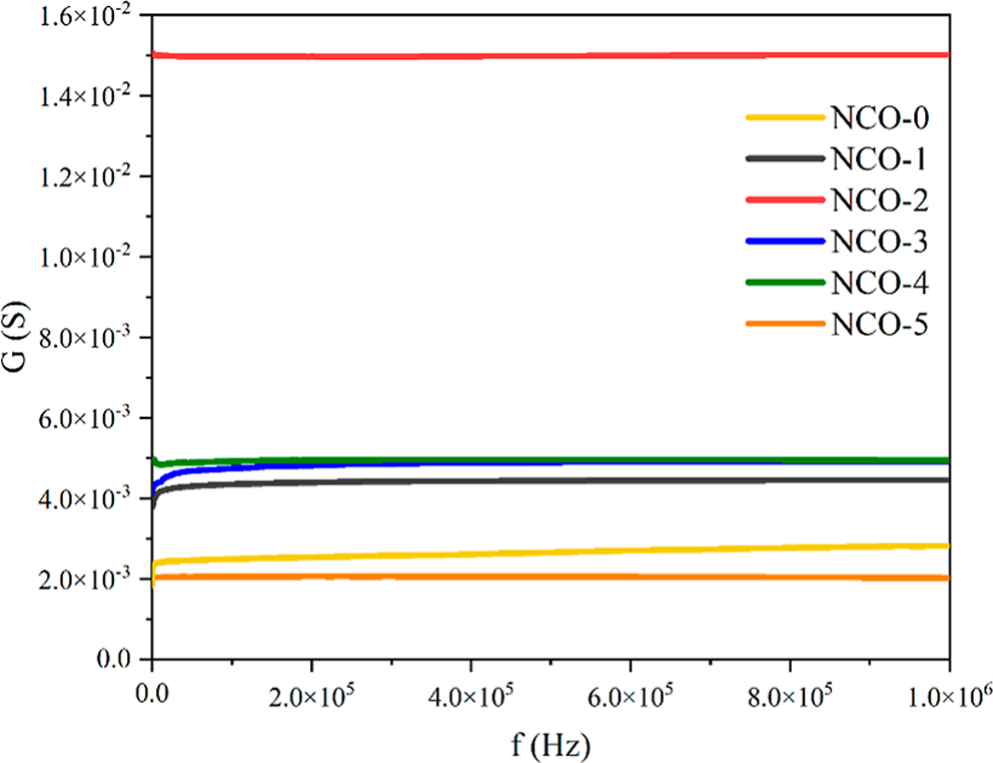
Conductance versus frequency plot of NCO nanostructures.

To determine the electrical properties of the NCO samples at room temperature, capacitance and conductance measurements were performed across a frequency range of 100 Hz to 1 MHz, as presented in Figures 9 and 10, respectively. Analysis of Figure 9 reveals that the capacitance values of all NCO samples are negative at low frequencies and show distinguishable variations among the samples. With increasing frequency, the capacitance of the NCO-0 sample stabilizes at a positive value in the nanofarad range, while the capacitance of samples NCO-1 to NCO-5, which were influenced by varying urea concentrations, stabilizes at negative values in the nanofarad range. This indicates that the presence of urea induces changes in electrical capacitance. Furthermore, at low frequencies, the NCO-2 sample exhibits a noticeably distinct capacitance compared to the other samples. This behavior is likely attributed to the porous fiber mesh-like nanoform characteristic of the NCO-2 sample. As a result, the negative capacitance phenomenon observed in all NCO samples at low frequencies is attributed in the literature to changes in the material’s polarization states,^60−62^ interfacial states,^63^ and the behavior of charge and minority carriers.^64,65^ In nanostructures synthesized from oxides of transition metal precursors such as Ni, Co, and Fe, it has been reported that a delay in current response to an AC bias voltage can occur due to electron hopping between nanoparticles.^66^ This phenomenon can lead to the observation of negative capacitance. Also the authors have previously detailed the occurrence of negative capacitance in NiCo_2_O_4_ nanomaterials in an earlier study on this field.^30^

The conductance values exhibited by the NCO samples in the frequency range of 100 Hz to 1 MHz are presented in Figure 10. It is observed that the conductance values of all samples are frequency independent. Additionally, the samples can be categorized into three distinct groups based on their conductance values measured in millisiemens: NCO-5 and NCO-0 form the first group, NCO-1, NCO-3, and NCO-4 constitute the second group, and NCO-2 represents the third group with the highest conductance values across all frequencies. The observed differences in conductance values among the NCO samples are attributed to variations in their morphologies, which result from the differentiation in crystallographic properties influenced by changes in urea concentration during the synthesis process. The influence of the nanomaterial’s morphology on conductance, as one of the key electrical properties of the material, has been demonstrated in various studies reported in literature.^67−69^

**Figure 11:**
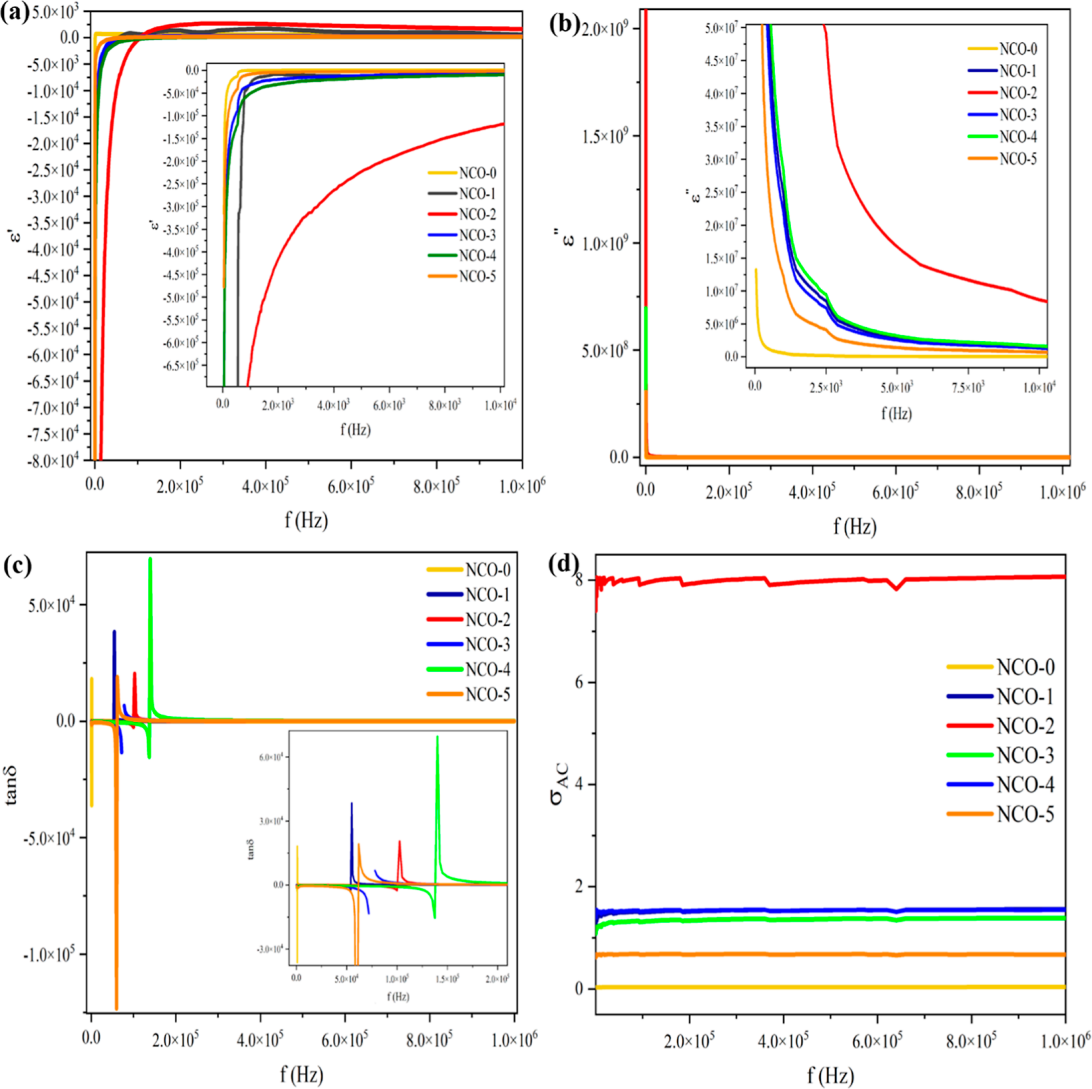
Dielectric parameters of NCO nanostructures: (a)ε′/*f* plots, (b) ε″/*f* plots, (c) tan δ/*f* plots, (d) σ_AC_/*f* plots.

The dielectric parameters, which are a crucial aspect of the material’s electrical characteristics, were determined through calculations based on the capacitance and conductance values measured from the bulk NCO samples. Figure 11a presents the graph depicting the variation of the real part of the complex dielectric constant with frequency, while Figure 11b illustrates the variation of the imaginary part of the complex dielectric constant with frequency. Figure 11c shows the variation of the dielectric loss tangent with frequency, and Figure 11d displays the variation of AC conductivity with frequency. The complex dielectric constant (ε*), which is one of the key electrical properties of NCO nanomaterials, represents a combination of the energy stored in the material (ε′) and the energy dissipated as loss (ε″). The equations used for calculating these parameters are provided in eqs 5–7). Here, *C* denotes the capacitance, *G* represents the conductance, *d* is the thickness of the bulk NCO sample, *A* is the surface area of the electrode contact, ω is the angular frequency, and ε_0_ is the permittivity of free space.^70^
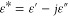
5
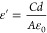
6
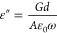
7

The negative capacitance phenomenon observed in all NCO samples is considered to be an inductive behavior and it is stated that the negative permeability (ε′) presented in Figure 11a is functionally equivalent to negative capacitance. Negative transmittance values are observed in the low-frequency region, gradually saturating as the frequency increases. This phenomenon is attributed to minority carriers, the behavior of free electrons, and the influence of interfacial states on the material.^71,72^ The saturation of permeability at high frequencies is linked to polarization mechanisms. Frequency-dependent changes in the polarization states of the sample are thought to result from the dielectric response of nanomaterials. It is noted that as the frequency increases, the inability of electric dipoles to keep up with the AC frequency initially reduces the dielectric constant value, which then reaches saturation.^73−76^

As shown in the scatter plot of the imaginary part of the dielectric constant (ε″) versus frequency in Figure 11b, a sharp decrease is observed in all NCO samples as the frequency increases. This behavior is attributed to the fact that, based on models such as Koop and Maxwell–Wagner in the literature, resistive grain boundaries play a more significant role than conductive grains at low frequencies.^77,78^ Consequently, the dielectric constant tends to exhibit higher values at lower frequencies.

The correlation between energy dissipation and energy accumulation in NCO samples is analyzed using the loss tangent (tan δ),^79^ as defined in eq 8.
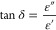
8

When the graph of the loss tangent versus frequency presented in Figure 11c is analyzed, distinct peaks are observed in the low-frequency region. These peaks occur when the applied frequency approaches the hopping frequency of the charge carriers. In the high-frequency region, the loss tangent is found to be zero, indicating that the NCO samples can store energy at high frequencies without any energy dissipation due to heating or other external factors. A loss tangent value of zero confirms that there is no energy loss in the system.^80,81^

The conduction mechanisms of the NCO samples were analyzed based on the electrical conductivity values (σ_AC_)^82^ under alternating current, as defined in eq 9.
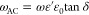
9

According to Jonscher’s power law,^83^ which is used to explain the frequency dependence of conductivity as presented in eq 10
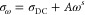
10

σ_ω_ represents the total conductivity of the material. The term σ_DC_ corresponds to the DC conductivity, while *A*ω^s^ represents the AC conductivity component. Here, *A* is a temperature-dependent constant, and s is a parameter that indicates the degree of interaction between charge carriers. When Figure 11d is analyzed, it is observed that the AC conductivity values of NCO samples exhibit minimal frequency dependence. This is because when the parameter s in eq 10, which represents an exponential function of frequency, is close to 0, the *A*ω^s^ value remains very small. Consequently, the DC conductivity becomes dominant in the material. In such cases, the ion mobility is explained by the correlated barrier hopping (CBH) mechanism.^84−86^ Additionally, the NCO-2 sample demonstrates higher AC conductivity values compared to the other samples across all frequency ranges. This behavior is attributed to the unique morphology of the NCO-2 sample, which consists of nanofibers containing hollows. Material synthesis parameters such as calcinating conditions, precursor molar ratios, reaction temperature, and reaction time directly influence the crystallographic structure of the final material. These structural changes, in turn, affect the material’s morphological characteristics. In the case of NCO samples, variations in crystallographic structure led to observable morphological differences. These differences affect the movement of the charge carriers, improving the efficiency of the bipolar hopping mechanisms, and consequently may change the AC conductivity values.^87,88^

In this study, all dielectric parameters of NiCo_2_O_4_ nanomaterials (NCO-0 to NCO-5) synthesized with different morphologies have been summarized at selected frequency values and presented in Table 3.

**Table 3:** Dielectric Parameters of NCOs with Different Nanoforms

	frequency	100 Hz				1 kHz				1 MHz			
	dielectric parameters	ε′	ε’’	tan δ	σ_AC_	ε′	ε’’	tan δ	σ_AC_	ε′	ε’’	tan δ	σ_AC_
sample name	NCO-0	–8.0 × 10^04^	5.5 × 10^06^	–8.4 × 10^01^	2.5 × 10^–2^	2.1 × 10^02^	5.9 × 10^05^	2.4 × 10^03^	3.1 × 10^–2^	5.7 × 10^02^	6.9 × 10^02^	1.2 × 10^00^	3.7 × 10^–2^
	NCO-1	–5.8 × 10^06^	2.4 × 10^08^	–5.1 × 10^01^	1.1 × 10^00^	–2.8 × 10^04^	2.5 × 10^07^	–9.8 × 10^02^	1.3 × 10^00^	5.9 × 10^02^	2.8 × 10^04^	2.6 × 10^01^	1.5 × 10^00^
	NCO-2	–2.6 × 10^06^	1.5 × 10^09^	–6.4 × 10^02^	6.9 × 10^00^	–6.5 × 10^05^	1.5 × 10^08^	–2.3 × 10^02^	7.8 × 10^00^	1.6 × 10^03^	1.5 × 10^05^	6.6 × 10^01^	8.0 × 10^00^
	NCO-3	–3.4 × 10^05^	2.1 × 10^08^	–7.4 × 10^02^	1.0 × 10^00^	–3.2 × 10^04^	2.2 × 10^07^	–7.1 × 10^02^	1.2 × 10^00^	2.3 × 10^02^	2.5 × 10^04^	9.6 × 10^01^	1.4 × 10^00^
	NCO-4	–3.8 × 10^05^	2.8 × 10^08^	–8.4 × 10^02^	1.3 × 10^00^	–5.0 × 10^04^	2.8 × 10^07^	–5.8 × 10^02^	1.5 × 10^00^	1.5 × 10^02^	2.8 × 10^04^	1.7 × 10^02^	1.5 × 10^00^
	NCO-5	–1.9 × 10^05^	1.2 × 10^08^	–7.7 × 10^02^	5.7 × 10^–1^	–8.1 × 10^03^	1.2 × 10^07^	–1.6 × 10^03^	6.5 × 10^–1^	1.8 × 10^02^	1.2 × 10^04^	6.9 × 10^01^	6.7 × 10^–1^

## 4 Conclusion

In this study, the hydrothermal synthesis method was employed to produce NiCo_2_ O_4_ nanostructures. To diversify the morphological forms of the NCO nanostructures, urea crystals were added to the hydrothermal solution at varying molar ratios. Morphological analyses revealed that urea significantly influenced the nanoform of NCO. Furthermore, crystallographic and chemical bonding analyses confirmed that the synthesized samples exhibit high-quality spinel cubic NiCo_2_ O_4_ structures consistent with the literature and free from impurity elements.

The NCO samples, diversified by the effect of urea, were compared against the reference NCO-0 sample, which was synthesized without urea, based on the results of all analyses. To evaluate the electrical characteristics of the NCO samples, bulk samples were prepared, and capacitance and conductance measurements were performed at room temperature across a frequency range of 100 Hz to 1 MHz. The measurements revealed that all NCO samples exhibited negative capacitance at low frequencies. The impact of this negative capacitance phenomenon on the dielectric parameters of the material was elucidated through calculations.

Also, this study is the first to demonstrate, through detailed discussions and findings, that variations in the nanoforms of NCO samples lead to significant differences in their electrical characteristics. As a result, it contributes to literature by highlighting the potential of this material for future technological innovations.
