# Supplementary material for: Impact of Diverse Nanostructure Forms of NiCo2O4 Bulk Ceramics on Electrical Properties
Source: ACS Omega. 2025 Apr 22;10(17):17875–86. doi: 10.1021/acsomega.5c00708 (PMC12059917; doi:10.1021/acsomega.5c00708)
Supplement: Supplementary file 1 — ao5c00708_si_001.pdf [file ao5c00708_si_001.pdf]

Impact of Diverse Nanostructure Forms of $NiCo_2O_4$ Bulk Ceramics on Electrical Properties

Orhun Dos [a,b,*] , Sukru Cavdar [c]

[a]Department of Advanced Technologies, Graduate School of Natural and Applied Sciences, Gazi University, Ankara, 06530, Türkiye
[b]Alparslan Defence Sciences and National Security Institute, National Defence University, Ankara, 06530, Türkiye
[c]Department of Physics, Faculty of Science, Gazi University, Ankara, 06530, Türkiye

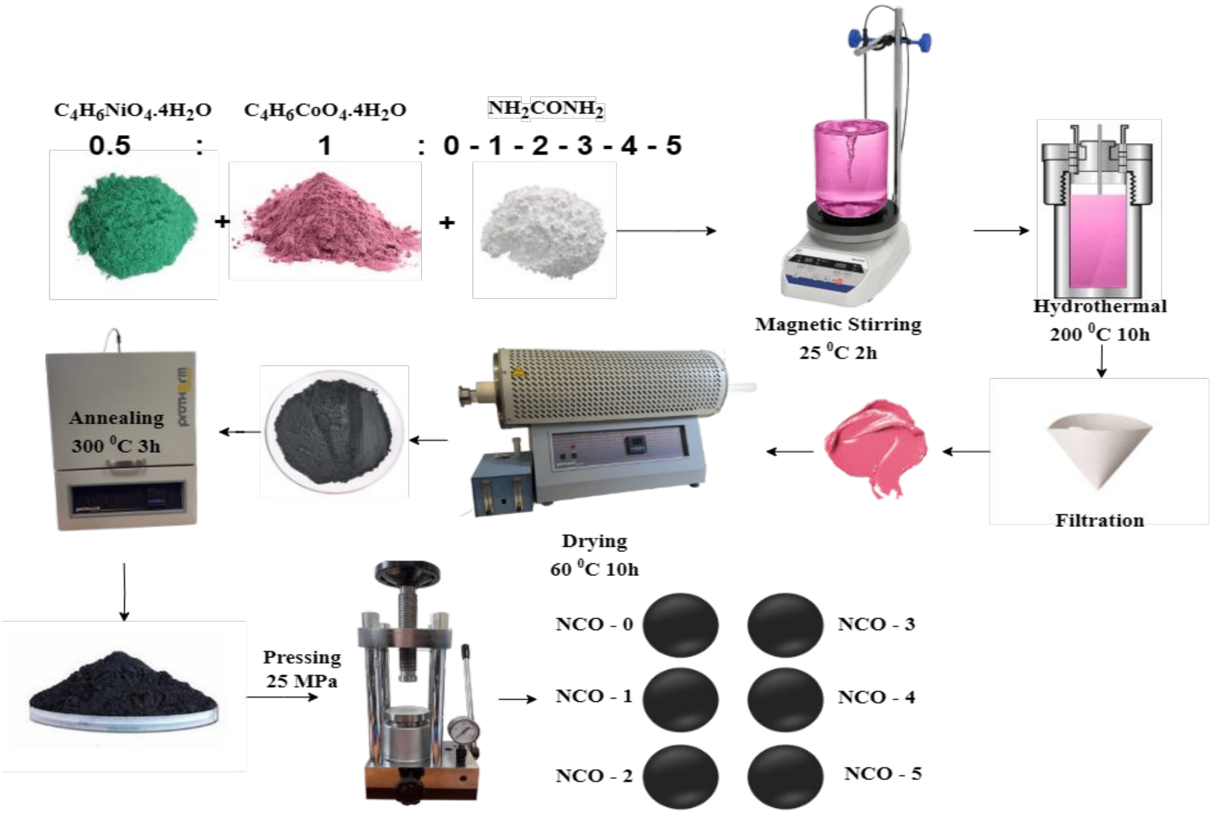

Figure S1. Experimental scheme of NCO production in hydrothermal method.

* Corresponding author.
E-mail address: orhun.dos@gazi.edu.tr, (O. Dos).

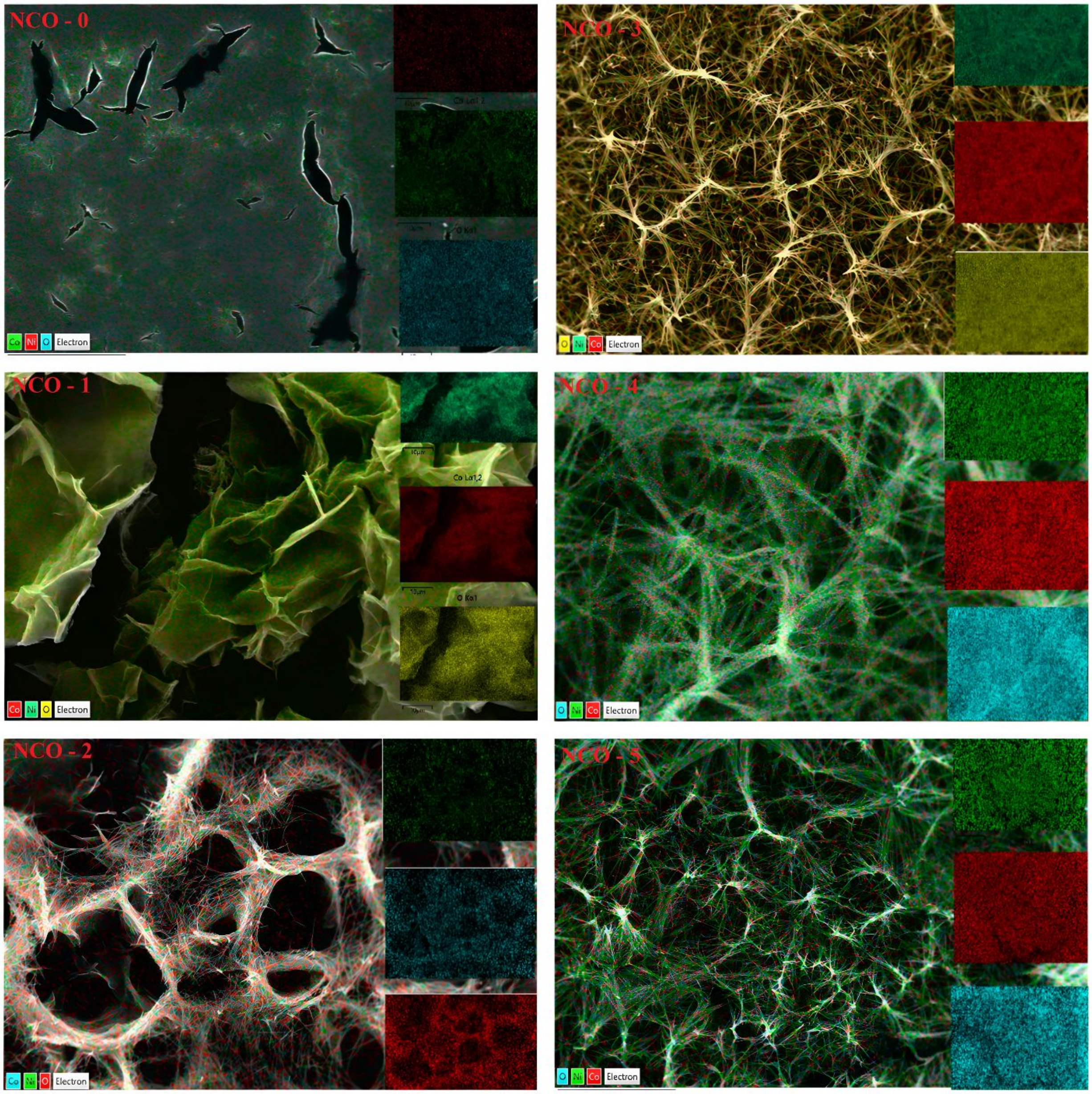

Figure S2. Elemental mapping images of NCO nanostructures.
